# Observations on the Oleates

**Published:** 1886-03

**Authors:** H. W. Stelwagon

**Affiliations:** Philadelphia


### Observations on the Oleates.

BY DR. H. W. STELWAGON, PHILADELPHIA.

In regard to the chemistry and preparation of the various oleates, both as to their manufacture by the direct combination of the acid with the base and by double decomposition, almost, if not, entirely, as much can be found in the English translation of Gmelin's Handbook of Chemistry, published 1866, as in the writings of the past several years.

Of all the oleates, those of mercury, zinc, bismuth, and lead, have a place in the treatment of diseases of the skin, and in view of their costliness, the seeming unavoidable frequency of badly made preparations, the disagreeable oleic acid odor, and the irritation so frequently observed following their use, it is probable that of these four the mercuric oleate promises to retain a permanent value. This last is especially valuable in ringworm of the scalp, but for inunctions in the treatment of syphilis it is of doubtful utility, as it is questionable whether it is absorbed.

Oleate of copper, which has been so highly recommended for ringworm of the scalp, is not comparable in that affection to oleate of mercury, or to tar and sulphur preparations.

Dr. Wigglesworth had practically renounced all oleates except the oleates of zinc, lead, and mercury, as parasiticides.

Dr. Duhring had employed the oleate of copper in varying strength in thirty or forty obstinate cases of ringworm, but it seemed to exert no influence whatever. As to its efficiency in acuter forms of ringworm, he could not speak.

Dr. Hardaway had almost entirely discarded the oleates. In some recent cases, the oleate of copper has seemed to be successful, but in chronic cases it entirely failed.

